# Assessment of Hospitalized Patients’ Awareness Regarding Food for Special Medical Purposes

**DOI:** 10.3390/nu18050808

**Published:** 2026-03-01

**Authors:** Aleksandra Raczyńska-Holińska, Teresa Leszczyńska, Piotr Skotnicki, Anna Spólnik, Aneta Koronowicz

**Affiliations:** 1 Department of Human Nutrition and Dietetics, Faculty of Food Technology, University of Agriculture in Krakow, al. Mickiewicza 21, 31-120 Krakow, Poland; teresa.leszczynska@urk.edu.pl (T.L.); anna.spolnik@student.urk.edu.pl (A.S.); 2 Department of Surgery with a Sub-Department of Oncological Surgery, Independent Public Health Care Facility in Bochnia “District Hospital” Named After Blessed Marta Wiecka, ul. Krakowska 31, 32-700 Bochnia, Poland; pskotnicki@vp.pl

**Keywords:** foods for special medical purposes, malnutrition, nutritional treatment, hospitalized patients, oral nutritional supplements

## Abstract

(1) Background: Malnutrition increases the risk of complications, prolongs the period of hospitalization, worsens the results of treatment, and increases the costs of hospital stay. Patients’ lack of knowledge on how to cope with it may increase the occurrence of these unfavorable consequences. The aim of this study was to assess hospitalized patients’ awareness of foods for special medical purposes (FSMP) and to determine the perception of the dietitian’s role in the hospital treatment process. (2) Methods: The survey was conducted among patients hospitalized in one of the hospitals in the Małopolska region. The sample consisted of 100 respondents. Participation in the research was anonymous and voluntary. The author’s survey contained 14 closed- and open-ended questions. The answers were single or multiple choice. A knowledge test was used to determine the level of awareness among respondents. The maximum score was 8. Appropriately selected tests were applied to the collected data, such as Spearman’s correlation, Shapiro–Wilk’s normality test, and Levene’s and Mann–Witney’s tests. The level of statistical significance was assumed to be *p* ≤ 0.05. (3) Results: Respondents were most familiar with the term Nutridrink (68%). Only 66% declared they knew what foods for special medical purposes were used for. Most were unfamiliar with the concept of immunomodulatory ingredients. Statistically significant correlation was found between age and knowledge. Older patients achieved lower scores (rho = −0.32, *p* = 0.001). No statistical significance was found between sexes or comorbidities and knowledge on the discussed topic. A dietitian was pointed out as the expert in selecting FSMP (78.6%). The findings indicate that that 87% of respondents believe that FSMP consumption may be beneficial for nutritional status. (4) Conclusions: The results indicate limited knowledge among hospitalized patients about foods for special medical purposes. The role of dietitians in the treatment process is highly valued by respondents. The study results suggest that educational initiatives in hospitals may be relevant to increasing patient awareness. Potentially, such initiatives could increase the effectiveness of nutritional therapy and preventive measures aimed at improving patient nutritional status.

## 1. Introduction

One of the many problems faced by hospitalized patients, especially the elderly, is malnutrition [1]. Malnutrition, as defined by the WHO, ASPEN, and ESPEN, is an imbalance between the supply and utilization of nutrients. It can involve both deficiency and an excess of nutrients, as well as diet-related diseases. Excessive nutrient intake leads to overweightness and obesity. These are diseases that cause metabolic disorders such as hyperlipidemia and hyperglycemia. Attention should be paid to substances secreted by adipose tissue (in people with excessive hypertrophy) such as leptin, resistin, or TNF-α, other cytokines and hormones. These substances act through many mechanisms, contributing to insulin resistance, improper appetite regulation, and increased proinflammatory cytokine secretion. Chronic excessive energy intake and visceral obesity can lead to type 2 diabetes, fatty liver disease, cardiovascular disease, and cancer [2,3]. The condition of sarcopenic obesity is often forgotten, where despite the increase in fat tissue, there is a decrease in muscle mass and strength [4]. Malnutrition is associated with a poorer prognosis, an increased risk of complications, and prolonged hospitalization [2,3,5]. It is a clinical condition resulting from poor nutrition or a disease that causes digestive and malabsorption disorders or symptoms that negatively affect appetite, resulting in reduced intake of micro- and macronutrients [6,7]. It is estimated that up to 50% of hospitalized patients may suffer from disease-related malnutrition, and 30% of patients will develop malnutrition during their hospital stay [8,9]. According to a review by Salari et al. [10], malnutrition among older adults worldwide is 18.6%. Malnutrition results in numerous adverse changes in the body, such as changes in body composition (loss of muscle mass and fat tissue) and deterioration of muscle function, including cardiac, renal, and respiratory muscle functions [10]. Gastrointestinal function regresses through impacts on the functions of the pancreas, intestinal villus, and colon, which can cause diarrhea and further impairment of digestion and absorption [6]. Malnourished patients have prolonged hospital stays and a higher risk of mortality, poorer response to treatment, impaired wound healing and immune function, and prolonged recovery time after procedures [11,12]. Elderly people and those with underlying health conditions (oncology, neurological, and critically ill patients) are particularly at risk [9,11]. A key approach to preventing malnutrition is proper assessment of nutritional status, which allows for early detection and mitigation of its progression and negative consequences. Effective tools include scales that include questions about weight loss over a selected period, current body weight, existing diseases, and food consumption.

If malnutrition is detected or a patient is at high risk, appropriate intervention should be initiated. It has been estimated that investing USD 1 in nutritional therapy will yield a USD 54 return on investment [13]. Depending on the needs and capabilities, dietary fortification with ONS, enteral nutrition, or parenteral nutrition is used, according to the ESPEN protocol [14]. If the patient is unable to consume adequate amounts of food, a dietary consultation is recommended to establish an oral diet, possibly supplemented with oral nutritional supplements (ONS). ONS are food for special medical purposes (FSMP), preparations that allow for the easy delivery of energy and protein in a small volume. Depending on the disease state, selected preparations may be enriched or omitted with certain essential ingredients. ONS can be classified in various ways. There are hypocaloric (0.5–0.9 kcal/mL), isocaloric (0.9–1.2 kcal/mL), and hypercaloric (1.3–2.4 kcal/mL) classifications. Based on their ingredient content, they are classified as high-energy, high-protein, and mixed [15]. Proteins can contain varying degrees of hydrolysis, including polymeric, oligomeric, and monomeric. Many different types allow them to be tailored to specific diseases. Formulations with reduced monosaccharides and saccharose contents, a low glycemic index, and added dietary fiber are intended for diabetics. In liver diseases at risk of encephalopathy, formulas with added branched-chain amino acids (BCAAs), fiber, and medium-chain triglycerides (MCTs) are recommended. For chronic kidney disease, hypercaloric ONS with reduced protein content (approximately 6% of energy) and electrolytes are used [16]. Immunonutrition is the supply of selected ingredients that modulate the immune response and inflammatory processes [17]. These preparations contain omega-3 fatty acids, selected amino acids such as glutamine and arginine, nucleotides, and prebiotic fiber. Formulas recommended for patients with difficult-to-heal wounds, ulcers, burns, and pressure sores contain arginine, collagen, and fatty acids, which stimulate repair processes and modulate the immune response [18,19]. For patients requiring dialysis, high-energy and high-protein ONS are used. For individuals with cow’s milk protein allergies and who are vegan, a formula based on plant proteins (soy) is recommended. Single-ingredient products such as protein, fiber, and thickeners are also available [16].

A lot of scientific data indicate that nutritional support through the use of ONS, along with oral diet fortification, can be crucial as an intervention against malnutrition and disease-related malnutrition [20,21,22,23,24,25]. The use of ONS for at least 7 days in patients in the preoperative period for gastrointestinal procedures reduces the incidence of infections and the length of hospitalization [20]. A literature review by Tangvik et al. [21] examined the effects of ONS supplementation in patients with dementia on daily dietary intake, nutritional status, and cognitive and functional functions, focusing on malnourished patients. ONS consumption had a positive effect on nutritional status and daily protein and energy intake, but no correlation was detected with improved cognitive or functional function. One study included 65-year-olds classified as at moderate-to-high-risk of malnutrition using the Malnutrition Universal Screening Tool (MUST). Participants were divided into two groups, control and treatment, for 180 days. The control group received two servings per day of ONS containing beta-hydroxy-beta-methylbutyrate, with dietary counseling, and the treatment group received two servings per day of a placebo supplement with dietary counseling. The results clearly established that ONS supplemented with HMB and vitamin D, along with dietary counseling, significantly improved nutritional and functional outcomes in older adults at risk of malnutrition, compared with placebo supplementation combined with dietary counseling [12]. Liu et al. [22] analyzed 22 studies of ONS consumption in patients undergoing hemodialysis (HD) or peritoneal dialysis (PD) who were at high risk of malnutrition due to their disease. The ONS group had significantly higher serum albumin levels, body mass index (BMI), and handgrip strength (HGS) from the beginning to the end of the intervention. No significant differences were observed between groups in terms of lean body mass, phase angle, C-reactive protein concentration, and serum phosphorus and potassium levels. The effectiveness of ONS in elderly patients with anorexia has also been assessed. Their use has been shown to improve appetite to some extent and to have a beneficial effect on body weight and food intake. Researchers also suggest a reduction in the incidence of pressure sores and diarrhea, as well as lower treatment costs, but these data should be comprehensively studied [23]. Benefits of ONS consumption have also been observed in malnourished children in developing countries, particularly on growth and weight gain. Adequate nutritional status in children characteristically improves motor and cognitive function in adolescents [24]. Baldwin et al. [25] suggest that the effects of ONS consumption in selected patient groups should be further investigated, given the unclear benefits of their use, based on 22 literature reviews [25].

There are studies assessing respondents’ knowledge of nutritional knowledge, but the number seems limited and targeted at different groups. A study by Dünya et al. [26] assessed the level of knowledge among nurses in the intensive care unit. A positive attitude toward nutritional care was found, but knowledge and quality in this area were insufficient [26]. Shakhshir and Alkaiyat described an assessment of the Modified Knowledge–Attitude–Practice (M-KAP) among nurses. (The entire group was 405.) The findings indicate insufficient knowledge and attitudes that do not support practical action. The researchers emphasize the need to employ nutrition specialists in hospital care [27]. As indicated, medical personnel often demonstrate gaps in nutritional competence, so the results of the study on insufficient patient awareness seem equally valid and relevant. Studies assessing the direct relationship between respondents and knowledge of FSMP are limited; further analysis of awareness among different respondent groups in this area is needed. It is crucial that both healthcare professionals and patients are aware of the potential benefits of using these supplements. Patients often show reluctance to use these supplements due to their non-conventional nature and difficulty persuading them to supplement their diet, despite difficulties with swallowing, chewing, or appetite.

The aim of this study was to assess hospitalized patients’ awareness of foods for special medical purposes (FSMP) and to determine the perception of the dietitian’s role in the hospital treatment process.

## 2. Materials and Methods

The survey was conducted among patients hospitalized in one of the hospitals in the Małopolska region. The sample consisted of 100 respondents. Participation in the research was anonymous and voluntary. The survey included patients hospitalized in the internal medicine and surgery department with an oncological sub-department; the condition excluding participation in the study was the patient’s lack of awareness or refusal. No data were obtained on education or place of residence, but respondents provided information on age, sex, and comorbidities. The author’s survey contained 15 closed- and open-ended questions, 14 of which were included in this manuscript. One question was rejected due to insignificant responses. The answers were single or multiple choice. The authors developed the questionnaire, and it was not formally validated before the study began. Participant characteristics are presented in Table 1.

Some respondents did not answer the questions regarding age and sex, hence the variable number of respondents (*n* = 98 or 99).

**Figure 1:**
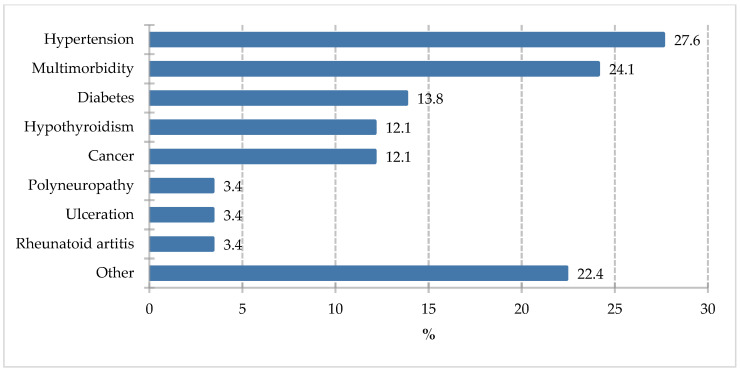
Comorbidities of respondents.

Respondents were also asked about comorbidities, and the answers are presented in Figure 1.

The most common medical conditions reported by respondents included hypertension (27.6%) and multiple morbidities (24.1%). Specific medical conditions included diabetes (13.8%), hypothyroidism (12.1%), and cancer (12.1%).

### Statistical Analysis

A knowledge test was used to determine the level of awareness among respondents. The maximum score was 8. The results were interpreted as follows: <25%—lack of knowledge, <50%—low level of knowledge, <75%—average level of knowledge, and >=75%—high level of knowledge. The interpretation of the knowledge level test was developed on the basis of literature and research on the knowledge of the respondents [28,29,30]. A statistical analysis was conducted based on the data obtained. The Spearman’s rank correlation coefficient was used to determine the direction and strength of the relationship between age and knowledge of food for special medical purposes. The following procedure was used to verify knowledge between sexes and the correlation between comorbidities and knowledge. The Shapiro–Wilk test was used to test for the normality of residual distribution, the Levene’s test to determine the homogeneity of variance between the compared groups, and the Mann–Whitney test to analyze the significance of differences in knowledge levels, taking into account sex or the presence of comorbidities. The assumed level of statistical significance in the analysis was *p* ≤ 0.05. Statistical analyses were performed using Statistica 13.3 (TIBCO Software Inc., Palo Alto, CA, USA).

## 3. Results and Discussion

**Figure 2:**
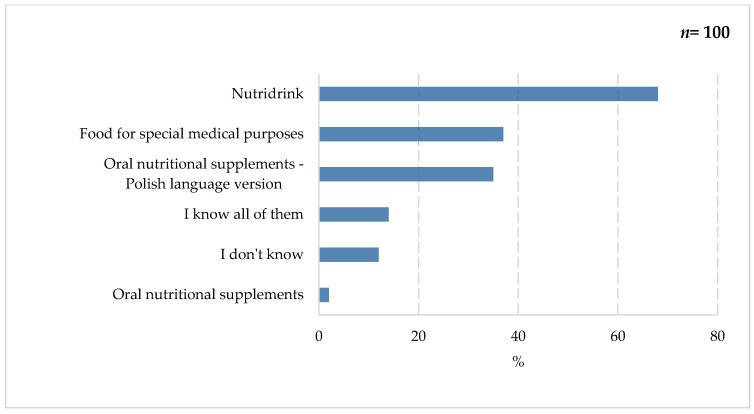
Percentage of respondents who answered the question: 100%. “Do you know the following concepts?”

As shown in Figure 2, the term “Nutridrink” was most frequently used by survey participants (68%). It is one of the trade names of a product from the leading brand FSMP on the Polish market. Despite respondents’ limited awareness of the product, the generic name ONS was still associated. Slightly fewer respondents indicated familiarity with “foods for special medical purposes” (37%) and oral nutritional supplements (35%). (The survey was conducted in Polish; therefore, the translated term and the English version were provided.) Less frequently selected were responses regarding familiarity with all of the terms (14%) and unfamiliarity with any of them (12%). The smallest proportion of respondents selected the ONS answer (the untranslated version) (2%).

**Figure 3:**
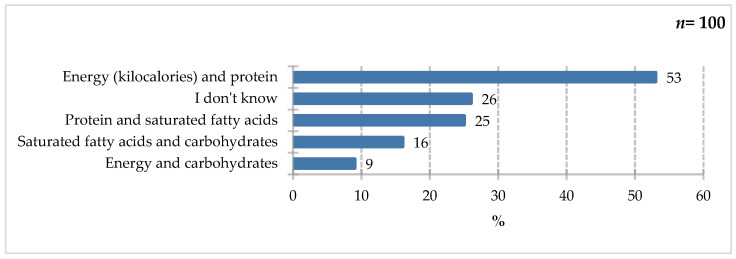
Percentage of respondents who answered the question: 100%. “Which ingredients would you pay attention to in your diet if you were to unintentionally lose significant weight in a short period of time?”

The largest group of respondents emphasized energy, kilocalories, and protein (53%), indicating an understanding of the role of individual macronutrients in the diet (Figure 3). Older adults should consume 1.2–1.5 g of protein/kg of body weight/day in acute or chronic conditions, and this intake should be increased to 2.0 g/kg/day in severe conditions or malnutrition. A total of 30 kcal/kg/day is the recommended energy requirement, but other factors should be considered, and recommendations should be individualized [14]. In a review of the ESPEN guidelines, the authors identified varying energy and protein requirements, depending on the disease state [31]. For example, in the intensive care unit, it is recommended to implement enteral nutrition for up to 48 h or parenteral nutrition between 3 and 7 days. If EN is not possible, the recommended energy requirement is 20–25 kcal/kg of body weight/day and 1.3 g protein/kg of body weight/day [32]. For geriatric patients, 30 kcal/kg of body weight/day is recommended. Oral nutritional support should provide at least 400 kcal, including at least 30 g of protein and at least 1.0 g/kg of body weight [14]. A significant proportion of respondents indicated protein and saturated fat (25%), as well as saturated fat and carbohydrates (16%). Nine percent of respondents emphasized energy and simple carbohydrates. Twenty-six percent of respondents declared they did not know the answer to this question.

**Figure 4:**
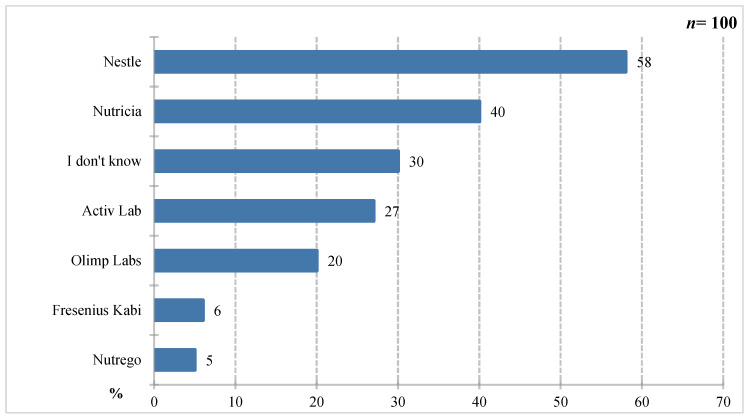
Percentage of respondents who answered the question: 100%. “Do you know companies that produce products for special medical purposes?”

According to the results in Figure 4, respondents most frequently cited Nestlé (58%), Nutricia (40%), and Activ Lab (27%). Olimp Labs (20%), Fresenius Kabi (6%), and Nutrego (5%) were mentioned less frequently. Thirty percent of respondents declared they were unaware of any company producing foods for special medical purposes. The high brand recognition of Nestlé and Nutricia may point to their visibility and the availability of a wide range of products, but no data or sources were collected that would include information on marketing exposure. At the same time, the fact that nearly one-third of respondents were unable to identify any manufacturer indicates the need for further education about foods for special medical purposes among patients. If patients do not recognize the brand, they are likely unfamiliar with the product range, which may limit the possibility of using specific products as the best choice for a given patient.

**Figure 5:**
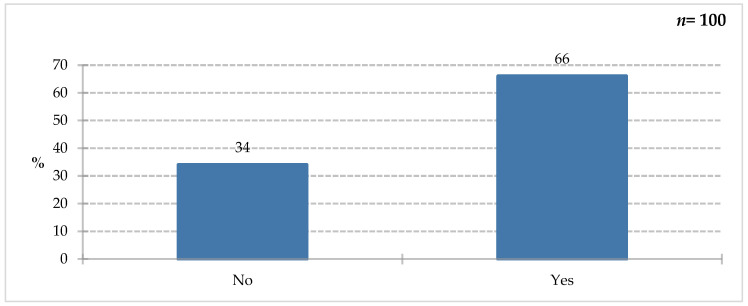
Percentage of respondents who answered the question: 100%. “Do you know what food for special medical purposes are used for?”

**Figure 6:**
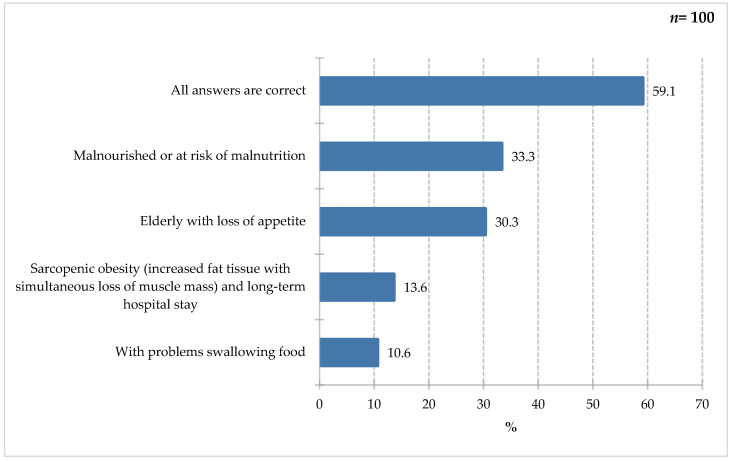
Percentage of respondents who answered the question: 68%. “You would use FSMP on people?” (Only people who answered yes to the previous question answered.)

Among all respondents, 66% declared they had knowledge of the purpose of using special medical devices (Figure 5). However, 34% of this study’s participants provided a negative answer. To verify the correctness of the affirmative answer, the following question (Figure 6) was asked: “You would use FSMP on people?” (Only people who answered yes to the previous question answered.)

Respondents most frequently indicated the use of special medical purposes in individuals with malnutrition or at risk of malnutrition (33.3%) and in elderly individuals with a lack of appetite (30.3%). Slightly less frequently selected were patients with sarcopenic obesity, characterized by increased body fat combined with loss of muscle mass and long-term hospitalization (13.6%), as well as individuals with swallowing difficulties (10.6%). Furthermore, 59.1% of respondents indicated that all of the above answers were correct, indicating a deeper understanding of the issue of malnutrition in this patient group, as all of the above factors are indications for oral dietary fortification, with ONS as the first step towards proper nutritional intervention. Indications for ONS use included the following:

- Perioperative period—patients undergoing major procedures, gastrointestinal surgery, and wound healing [33,34];
- Oncological diseases—insufficient oral intake and prevention of malnutrition [35];
- Elderly patients—individuals with sarcopenia, frailty syndrome, and reduced appetite [14];
- Nephrology, pulmonology, neurology, and psychiatry—debilitating, chronic diseases causing reduced intake, appetite disorders, and swallowing disorders [34].

**Figure 7:**
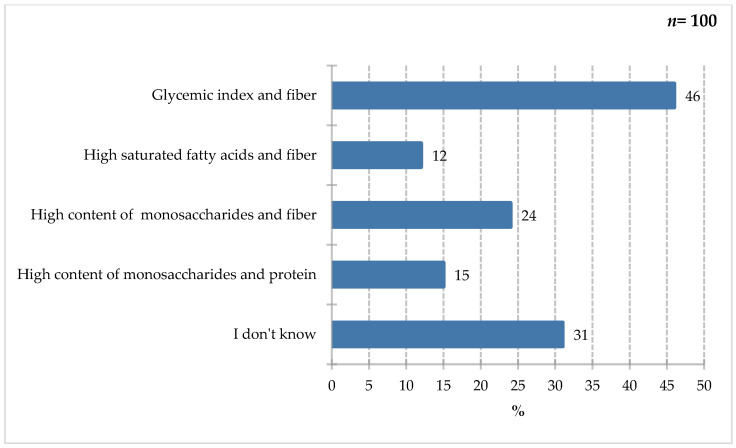
Percentage of respondents who answered the question: 100%. “Which ingredients would you pay attention to when choosing a product for people with diabetes?”

The results of Figure 7 indicate that respondents most frequently selected the glycemic index and fiber response (46%). Fewer participants indicated high simple sugar and fiber content (24%), high simple sugar and protein content (15%), and high saturated fat and fiber content (12%). Thirty-one percent of respondents declared a lack of knowledge in this area. Diabetes is a disease affecting a growing part of society. It often coexists with other conditions and carries complications, such as diabetic foot and prolonged hospital stays. Patients with the disease, as well as others at increased risk, should be aware of the ingredients to look out for, which will also facilitate the decision-making process of selecting ONS for patients with glycemic disorders. According to the Polish recommendations [36], it is necessary to pay attention to the glycemic load and index of a meal and to consume adequate macronutrients, including fiber-rich carbohydrates (minimum 25 g/day or 15 g of fiber/1000 kcal of diet) [36]. Limiting the consumption of simple sugars, especially added and free sugars, in favor of low-calorie sweeteners is crucial. Attention has also been paid to the supply and quality of fats, including limiting saturated fats to 10% of the diet’s energy value in favor of plant-based fats [36].

**Figure 8:**
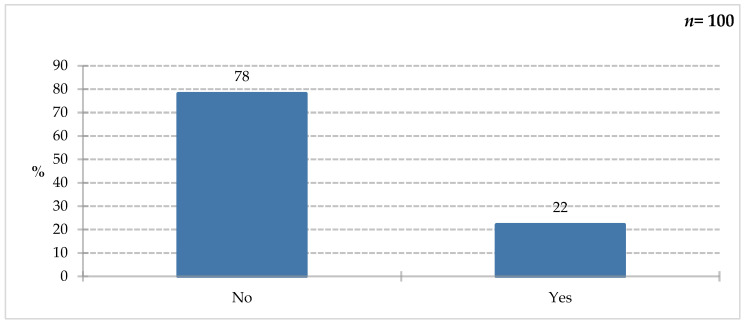
Percentage of respondents who answered the question: 100%. “Do you know any immunomodulatory ingredients that can improve the body’s immunity?”

**Figure 9:**
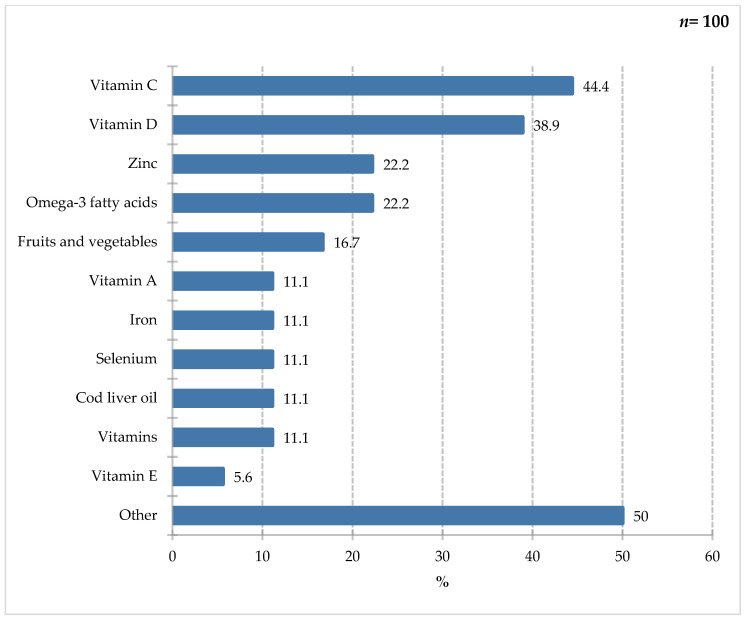
Percentage of respondents who answered the question: 18%. “If you answered YES in question 7, please specify which ingredients.”

The majority of respondents (78%) were unfamiliar with immunomodulatory ingredients; only 22% of survey participants declared they were familiar with them (Figure 8). To confirm their knowledge, they were asked to list them in the next question (Figure 9).

A large proportion of the responding group mentioned vitamin C (44.4%) and vitamin D (38.9%), with slightly smaller groups highlighting ingredients such as omega-3 fatty acids, zinc (22.2%), selenium iron, and vitamin A. Finally, the “other” category, selected by 50% of study participants, included less relevant responses such as elderberry and the aforementioned fruits or vegetables but also vitamin E, probiotics, and vitamin E, which fall into this category. Vitamin C, as a cofactor of many enzymes and one of the important antioxidants, can have immunomodulatory effects. Adequate dietary levels of vitamin C reduce C-reactive protein levels. By stimulating neutrophil migration, it improves wound healing. Studies indicate that vitamin C supplementation may shorten the duration of infection [34,37,38,39]. The action of vitamin D is widely described in literature. It influences the secretion of cytokines (IL-12, IL-2, and IFN-γ) by macrophages and regulates gene expression and cell differentiation [34,40]. There is a correlation between the activity of B and T lymphocytes and antigen-presenting cells and vitamin D. It inhibits the secretion of C-reactive protein and IL-6 [41].

**Figure 10:**
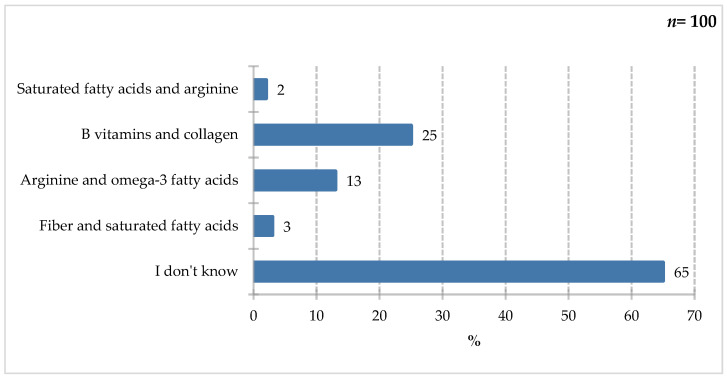
Percentage of respondents who answered the question: 100%. “Which ingredients would you recommend to people with difficult-to-heal wounds and pressure sores?”

As shown in Figure 10, respondents most frequently indicated B-group vitamins and collagen (25%). Less frequently selected were arginine and omega-3 fatty acids (13%), fiber and saturated fatty acids (3%), and saturated fatty acids and arginine (2%). A significant proportion of respondents (65%) declared they did not know the answer to this question. A literature review by Arribas-López et al. [42] indicated a positive effect of arginine and glutamine supplementation on wound healing or related parameters [42]. In turn, patients supplemented with arginine, vitamin C, and zinc showed a clinically significant improvement in pressure ulcer healing [43]. A study by Cheshmeh et al. [44] also found a beneficial effect of arginine on pressure ulcer healing. Arginine and its metabolites improve wound healing through metabolism and nitric oxide (NO) synthesis, but attention is also drawn to the addition of other components, such as an adequate supply of calories and protein [44,45]. Elahi et al. [46], in a review on the effects of fish oil, including omega-3 fatty acids, described various mechanisms and effects on the healing of stage 1 pressure ulcers. They indicated the formation of blood vessels in the ulcer, shortened hospital stays in surgical patients, and stimulation of cytokine activity [46]. In turn, in patients in the intensive care unit, the use of fish oil locally as dressings has a beneficial effect in preventing pressure ulcer development [47]. According to the review by Dospra et al. [48], supplementation with eicosapentanoic acid (EPA) in the postoperative period may contribute to reducing the risk of infection, supporting the wound healing process, and shortening the recovery time of patients [48].

**Figure 11:**
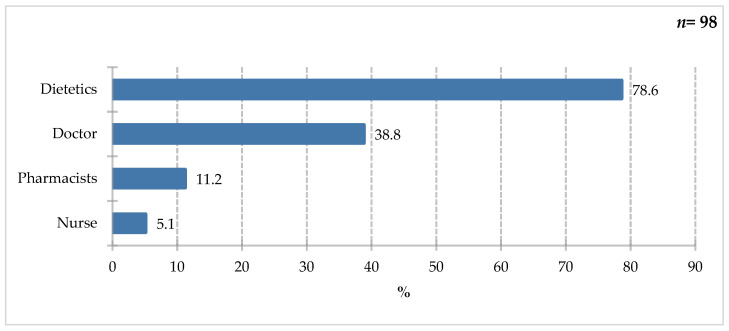
Percentage of respondents who answered the question: 98%. “Which specialist would you turn to for advice on the selection of FSMP?”

Survey participants most often cited a dietitian (78.6%) as the specialist they would seek advice from on selecting special medical supplies. Physicians (38.8%) and pharmacists (11.2%) were significantly less likely to be chosen. Nurses were cited by 5.1% of respondents (Figure 11). Although, by law, only a physician can prescribe ONS for a hospitalized patient, a dietitian can be an important part of the process. Patient responses also indicate a growing trust in this professional group.

**Figure 12:**
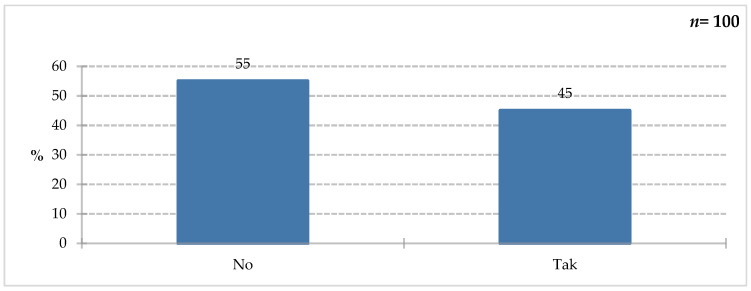
Percentage of respondents who answered the question: 100%. “Have you ever used food for special medical purposes during your hospitalization?”

Forty-five percent of respondents confirmed that they had experience with this type of food during their hospital stay (Figure 12).

**Figure 13:**
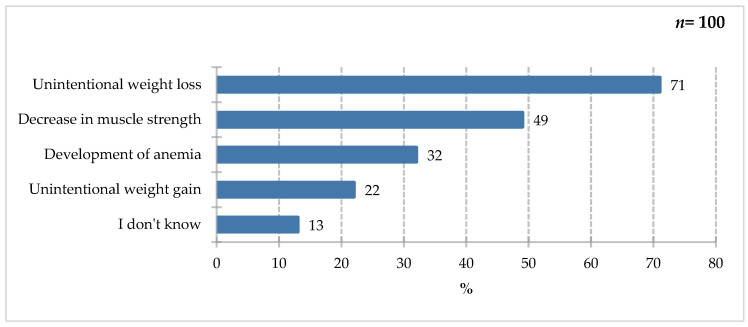
Percentage of respondents who answered the question: 100%. “Which of the following symptoms would you notice as risk factors for malnutrition?”

The largest share of study participants reported unintentional weight loss (71%). Decreased muscle strength (49%) and the development of anemia (32%) were also frequently mentioned. Unintentional weight gain (22%) and lack of knowledge on the subject (13%) were less frequently mentioned (Figure 13). Underweightness in adults is defined as a BMI below 18.5. Consequences of insufficient dietary intake include impaired function, negative impact on muscle mass and fitness, sarcopenia, and the development of anemia. Unintentional weight gain can lead to overweightness and obesity, but it does not correlate with an increased risk of underweightness, which does not exclude the occurrence of sarcopenia and qualitative malnutrition in excess body weight [49].

**Figure 14:**
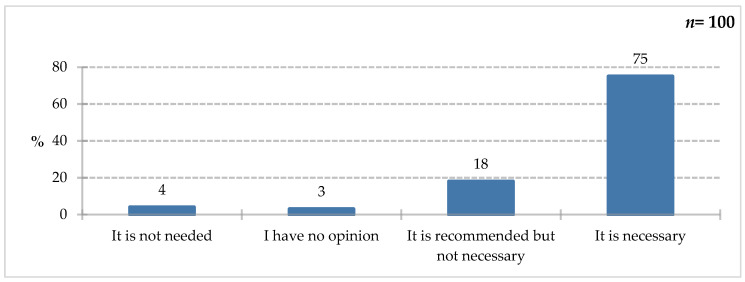
Percentage of respondents who answered the question: 100%. “How do you assess the need for cooperation with a dietitian in hospital wards?”

Respondents most often believed that collaboration with a dietitian in hospital wards was necessary (75%). It was recommended, but not essential, according to 18% of respondents; only 4% declared it unnecessary, while 3% had no opinion on the matter (Figure 14). It is encouraging that patients are beginning to understand the role of nutrition and the need for interdisciplinary collaboration. Dietitians in hospitals play a role as important as other medical professionals, assessing nutritional status, providing nutritional education to patients, fortifying a natural diet, and collaborating with the interdisciplinary team to determine nutritional treatment. According to a 2018 report, the role of dietitians in hospitals is still misunderstood. In audited facilities, nutritional education was lacking, with each dietitian serving between 76 and 740 patients, and additional duties unrelated to their competencies were assigned, such as warehouseman, kitchen assistant, or archivist [50]. This may be due to the lack of regulations regarding the scope of duties and competences of the dietitian. Bator assessed the nutritional knowledge of healthcare workers, and the results were clear: the staff’s knowledge was insufficient, and the dietitian should be responsible for providing nutritional education to patients [51]. Folwarski and Wernio clearly described the benefits of employing a dietitian in hospitals, such as improved detection of malnutrition and shorter hospital stays, as well as significant savings due to the accuracy of nutritional intervention tailored to the patient’s condition and needs [13]. Mogiłko and Zarzeczny [52], in turn, emphasized the dietitian’s important role in the prehabilitation process as one of the new but crucial tasks in patient care. This includes the selection of individualized nutritional recommendations, assessment of nutritional status, and continuous monitoring of outcomes [52].

**Figure 15:**
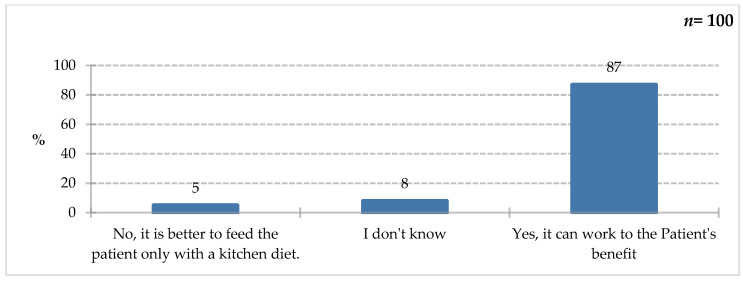
Percentage of respondents who answered the question: 100%. “Do you think that the use of FSMP purposes can be an important measure to improve or maintain the nutritional status of hospitalized patients?”

The vast majority of respondents (87%) agreed that the use of foods for special medical purposes can be a significant step towards improving or maintaining the nutritional status of hospitalized patients, pointing to the potential benefits for these patients (Figure 15). As previously indicated, ONS are used in many medical conditions. It is worth noting that 13% of respondents either lacked knowledge on this topic or believed that a traditional kitchen diet is sufficient. This indicates the need for educational initiatives.

In the knowledge test regarding foods for special medical purposes, participants scored an average of 3.90 (SD = 1.867) points out of 8 possible points. The mean percentage score was 48.75% (SD = 23.33). The interpretation of participants’ knowledge is presented in the graph (<25%—lack of knowledge, <50%—low level of knowledge, <75%—average level of knowledge, ≥75%—high level of knowledge).

**Figure 16:**
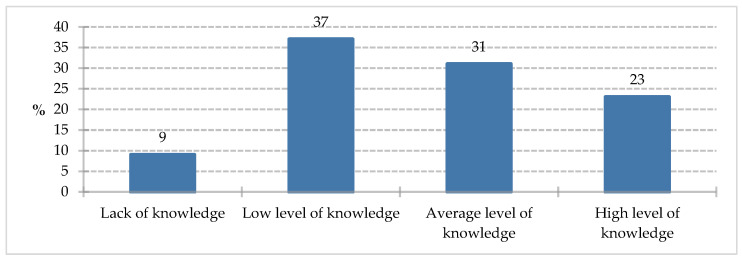
Distribution of knowledge among study participants.

Respondents most often demonstrated a low level of knowledge in the test (37%). An average level of knowledge was recorded for 31% of study participants. A high level of knowledge was achieved by 23% of respondents, and a lack of knowledge in the area under study was noted by 9% of participants (Figure 16). The results obtained in the study may suggest that patient knowledge of foods for special medical purposes is varied and also limited. Considering the important role of nutrition in modern medical therapy, the average score of approximately half the points may be considered as requiring further analysis and research.

The results of the statistical analysis are presented in Table 2. 

To determine the direction and strength of the relationship between age and knowledge about specialty foods, Spearman’s monotonic correlation coefficient was used. The results of the Spearman’s rho correlation analysis indicated a significant correlation between age and knowledge about special-purpose foods, with rho = −0.32, *p* = 0.001, and n = 98. The strength of the correlation turned out to be moderate, and the direction of the correlation turned out to be negative. With increasing age, the level of knowledge about special-purpose foods decreased significantly.

The observed negative association between age and knowledge of foods for special medical purposes may have clinical significance, particularly in the context of geriatric care. It should be noted that this was an exploratory study, and the phenomena described requires confirmation in further research. Older patients are at the highest risk of disease-related malnutrition, sarcopenia, and functional decline while simultaneously demonstrating lower awareness of available nutritional interventions. These findings demonstrate the potential need for targeted, age-adapted nutritional education strategies in hospital settings, with particular emphasis on geriatric patients, for whom simplified communication, repeated counseling, and close cooperation with dietitians may be considered as important elements of effective malnutrition prevention and lifecare. Such an approach aligns with the concept of lifecare, emphasizing not only the provision of nutritional support, but also patient-centered education as an integral component of comprehensive malnutrition management in older adults.

The Shapiro–Wilk test was used to assess the normality of the distribution; the results indicated a significant deviation from the normal distribution (W = 0.87; *p* = 0.019 and W = 0.95 and *p* = 0.001). Levene’s test was used to assess the homogeneity of variance between groups. Both analyses, by sex and comorbidities, demonstrated a lack of homogeneity of variance. The Mann–Whitney U test revealed no statistically significant differences in knowledge levels between women and men or between individuals with and without comorbidities. The presented results and the lack of significant differences between different patient groups highlight a broader issue. An important point seems to be that even patients with chronic diseases who may potentially require the previously indicated nutritional interventions, including FSMP, are insufficiently educated about nutrition and possible interventions.

The considered research results and literature data indicate actions aimed at expanding and shaping the knowledge and awareness of hospitalized patients, as well as their medical caregivers, are becoming a priority. Particular emphasis on support and education should be directed at geriatric patients, with the message being simplified but effective and repeated.

This study has several significant methodological limitations. It included a relatively small number of respondents and did not include a prior power calculation, which may have led to the failure to detect some differences and increased the risk of bias. Interpretation problems were initially caused by questions that respondents answered multiple times. Potentially relevant variables such as education, economic status, and place of residence were also not considered. Other factors that could be of specific importance included: previous hospitalizations, prior contact with a dietitian, and prior FSMP consumption. Multivariate statistical modeling was not performed. Therefore, the findings should be interpreted with appropriate caution. The exploratory nature of the study and the resulting conclusions may help indicate what should be considered in future studies on this topic. Considering multicenter populations, validating the questionnaire, and patients’ functional competence and nutritional status seem important.

## 4. Conclusions

This study demonstrated a statistically significant negative association between knowledge and age. There were no statistically significant differences in knowledge level between sexes or in knowledge level between patients with and without comorbidities. Overall, the results suggested limited patient knowledge of foods for special medical purposes, especially among the older population. These results highlight that educational initiatives in hospitals may be relevant. Due to the exploratory nature of the study, further multi-center studies with a validated tool are essential to confirm and extend the findings.

## Figures and Tables

**Table 1:** Basic characteristics of the respondents.

*n* (%)	*n*	99.
	99	Sex
53 (53.5%)		Female
46 (46.5%)		Male
	98	Age
19 (19.4%)		18–35
38 (38.8%)		36–55
25 (25.5%)		56–70
16 (16.3%)		over 70

**Table 2:** Statical analysis.

Analysis		Variable(s)	*N*	Statistic/Descriptive Value	*p*
Descriptive statistics	Knowledge	100		M = 3.9 ± 1.86; Me = 4.00; Min–Max: 0–7; Skewness = 0.10; Kurtosis = −1.08	-
	Knowledge Percentage	100		M = 48.75 ± 23.33; Me = 50.00; Min–Max: 0–87.50; Skewness = 0.10; Kurtosis = −1.08	-
Correlation analysis		Age-Knowledge	98	Rho = −0.32	0.001 ***
Descriptive statistics (sex)		Female (F)-Knowledge	53	M = 4.23 ± 2.06; Me = 5.00; Min–Max: 0–7; IQR = 4.00	-
		Male (M)-Knowledge	46	M = 3.57 ± 1.55; Me = 3.00; Min–Max: 0–7; IQR = 2.75	-
Normality test (sex)		Knowledge	100	W = 0.97	0.019 *
Variance equality (sex)		Knowledge	99	F(1,97) = 4.14	0.045 *
Group comparison (sex)		Knowledge	99	U = 1465.00; r_rb_ = 0.20	0.081
Descriptive statistics (comorbidities)		No	42	M = 3.76 ± 1.70; Me = 4.00; Min–Max: 0–7; IQR = 2.00	-
		Yes	58	M = 4.00 ± 1.98; Me = 3.50; Min–Max: 0–7; IQR = 4.00	-
Normality test (comorbidity)		Knowledge	100	W = 0.95	0.001 ***
Variance equality (comorbidity)		Knowledge	100	F(1,98) = 4.06	0.047 *
Group comparison (comorbidity)		Knowledge	100	U = 1157.50; r_rb_ = −0.05	0.671

Explanation of abbreviations: N—number of valid observations, M—mean, Me—median, Min.—minimum value, Max.—maximum value, * *p* < 0.05, *** *p* ≤ 0.001 indicate levels of statistical significance.

## Data Availability

The original contributions presented in this study are included in the article. Further inquiries can be directed to the corresponding authors.
